# Supplementary material for: Content-rich biological network constructed by mining PubMed abstracts
Source: BMC Bioinformatics. 2004 Oct 8;5:147. doi: 10.1186/1471-2105-5-147 (PMC528731; doi:10.1186/1471-2105-5-147)
Supplement: Additional File 2 — The original results of the above study (non-essential files are deleted to keep the file size under the limit set by BMC bioinformatics). [file 1471-2105-5-147-S2.bz2 › chilibotAdditionalFile2/dip20/337ID8621492E4139/html/LTA_LTB.html]

 


 **LTA** and **LTB** 
  
Found 137 abstracts in PubMed, retrieved 20.  
 

 What does Google say? 
 PDF only 
| .edu only 

---

**Interactive relationship** (e.g. stimulation, inhibition, etc)

**Neutral relationship**- The endogenous  **LTA**  4 hydrolase form the  **LTB**  4 from  **LTA**  4 in erythrocytes, platelet, endothelial or smooth muscle cells.  Ref: 12208150 Med Hypotheses, 2002
- **LTA**  4 is further metabolized to either LTC 4 or  **LTB**  4 by the enzyme LTC 4 synthase or  **LTA**  4 hydrolase, respectively.  Ref: 12021253 J Clin Invest, 2002
- LT consists of soluble LT alpha  [ **LTA** ]  3, which binds to TNF R1 and TNF R2, and membrane LT alpha  [ **LTA** ]  1 beta 2, which binds to LT beta  [ **LTB** ]  R.  Ref: 12882833 Int Immunol, 2003
- Transplantation of lymphoid tissues from LT beta  [ **LTB** ]  mice, which have cervical and mesenteric lymph nodes, into LT alpha  [ **LTA** ]  mice, which do not, did not alter the incidence of CNS scrapie.  Ref: 11932402 J Virol, 2002
- Furthermore, LT alpha  [ **LTA** ]  mice had a higher incidence and shorter incubation period for developing disease following inoculation than did LT beta  [ **LTB** ]  mice.  Ref: 11932402 J Virol, 2002
- This observation previously has led to the suggestion that  **LTA**  4 produced by leukocytes may, in some cases, be delivered to other cell types before being converted into LTC 4 or  **LTB**  4.  Ref: 12021253 J Clin Invest, 2002
- Additional structure activity relationship studies around this structural class resulted in the identification of a series of alpha, beta, and gamma amino acid analogues that are potent inhibitors of the  **LTA**  4 hydrolase enzyme and demonstrated good oral activity in a mouse ex vivo whole blood  **LTB**  4 production assay.  Ref: 12139459 J Med Chem, 2002

**Non-interactive relationship** (e.g. studied together, co-existance, homology, etc.)

- The enzyme  **LTA**  4 hydrolase represents an attractive target for pharmacological intervention in these disease states, since the action of this enzyme is the rate limiting step in the production of  **LTB**  4.  Ref: 12139459 J Med Chem, 2002
- Lymphotoxin alpha  [ **LTA** ] , lymphotoxin beta  [ **LTB** ] , C C chemokine ligand CCL 21, CXC chemokine ligand CXCL 12, CXCL13, and CCL22 were increased in thyroids from autoimmune patients.  Ref: 12794165 J Immunol< MedlineTA>J Immunol, 2002
- Lymphotoxin alpha  [ **LTA** ]  and lymphotoxin beta  [ **LTB** ]  deficient mice differ in susceptibility to scrapie evidence against dendritic cell involvement in neuroinvasion.  Ref: 11932402 J Virol, 2002
- We used mice lacking Peyer s patches and or lymph nodes because of lymphotoxin alpha  [ **LTA** ]  gene deficiency or treatment in utero with lymphotoxin beta  [ **LTB** ]  receptor IgG and tumor necrosis factor receptor I 55 IgG fusion proteins.  Ref: 12466141 Am J Pathol, 2002
- The LT alpha  [ **LTA** ]  and LT beta  [ **LTB** ]  R mice failed to mount a virus specific cytotoxic T cell response.  Ref: 12882833 Int Immunol, 2003
- FDC generation and maintenance in germinal centers is dependent on lymphotoxin alpha  [ **LTA** ]  LT alpha  [ **LTA** ]  and LT beta  [ **LTB** ]  signaling components.  Ref: 11932402 J Virol, 2002
- ... the  **LTA**  4 hydrolase inhibitor SC57461A and the LT biosynthesis inhibitor MK 0591 inhibited AA but not  **LTB**  4 mediated PLD activation.  Ref: 12660228 J Leukoc Biol, 2003
- Further, it is possible that an as yet unidentified cell found more abundantly in LT alpha  [ **LTA** ]  than in LT beta  [ **LTB** ]  mice may assist in the amplification of scrapie infection in the periphery and favor susceptibility to CNS disease following peripheral routes of infection.  Ref: 11932402 J Virol, 2002
- Neither lymphotoxin alpha  [ **LTA** ]  nor lymphotoxin beta  [ **LTB** ]  receptor expression is required for biogenesis of lymphoid aggregates or differentiation of natural killer cells in the pregnant mouse uterus.  Ref: 12603600 Immunology, 2003
- There was relatively little demyelination associated with inflammation in LT alpha  [ **LTA** ]  and LT beta  [ **LTB** ]  R mice compared to susceptible SJL mice, suggesting the possibility that LT plays a role in mediating demyelination.  Ref: 12882833 Int Immunol, 2003
- Cyclosporin A blocks the expression of lymphotoxin alpha  [ **LTA** ] , but not lymphotoxin beta  [ **LTB** ] , in human peripheral blood mononuclear cells.  Ref: 12176893 Blood, 2002
- We report here that by the oral route, LT alpha  [ **LTA** ]  mice developed scrapie while LT beta  [ **LTB** ]  mice did not.  Ref: 11932402 J Virol, 2002
- This activity of LT depends on membrane LT alpha  [ **LTA** ]  1 beta 2 and not soluble LT alpha  [ **LTA** ]  3, since DA virus persisted and induced inflammatory demyelination in LT beta  [ **LTB** ]  R, but not TNF R1 or TNF R2, mice.  Ref: 12882833 Int Immunol, 2003
- Expression of cytokines TNF beta  [ **LTA** ]  and LT beta  [ **LTB** ]  preceded, and the expression of TGF beta1 followed chemokine upregulation.  Ref: 12828562 Scand J Immunol, 2003
- Functional studies in gene knockout and transgenic mice systems have shown that lymphotoxin alpha  [ **LTA** ]  and lymphotoxin beta  [ **LTB** ]  LT alpha  [ **LTA** ]  and LT beta  [ **LTB** ]  are of fundamental importance in peripheral lymphoid organ development, but it remains unclear what role these cytokines have to play in the adult immune response and in the pathogenesis of disease.  Ref: 12474234 J Pathol, 2003
- Expression of IL 1alpha, IL 1beta, IL 6, IL 10, IL 12, IFN beta, IFN gamma, LT beta  [ **LTB** ] , TGF beta, TNF a and TNF beta  [ **LTA** ]  mRNA levels in CRT MG human astroglioma cells upon Fas ligation was investigated using RNase protection assay RPA.  Ref: 11949822 J Neurooncol, 2002
- Particular alleles of the tumor necrosis factor gene cluster TNFA,  **LTA** ,  **LTB**  are known to be associated with peculiar clinical forms of sarcoidosis.  Ref: 12102613 Sarcoidosis Vasc Diffuse Lung Dis, 2002
- Samples homozygous at TNFA 308 and BAT1 intron 10 demonstrated identity between the African TNFA 308\*2 haplotype, the 8.1AH and the Asian diabetogenic 58.1AH HLA A33, B58, DR3 across a region spanning BAT1, ATP6G, IKBL,  **LTA** , TNFA,  **LTB** , LST 1 and AIF 1.  Ref: 12859597 Tissue Antigens, 2003
- To investigate LT alpha  [ **LTA** ]  beta s activities in lymphoid organogenesis, mice simultaneously expressing LT alpha  [ **LTA** ]  and LT beta  [ **LTB** ]  under rat insulin promoter II RIP control were compared with RIPLT alpha mice in a model of lymphoid neogenesis and with LT beta  [ **LTB** ]  mice.  Ref: 12732657 J Exp Med, 2003
- We investigated leukotriene A 4  **LTA**  4 hydrolase expression and  **LTB**  4 levels in oral epithelium in relation to the clinical disease manifestation and immunohistopathology and  **LTA**  4 hydrolase expression in cultured oral keratinocytes.  Ref: 12070603 Virchows Arch, 2002
- This study suggests that  **LTA**  4 hydrolase expression and elevated  **LTB**  4 levels in oral mucosal epithelium are integral parts of the induction and progression of chronic inflammatory reactions.  Ref: 12070603 Virchows Arch, 2002
